# Oleuropein, unexpected benefits!

**DOI:** 10.18632/oncotarget.15538

**Published:** 2017-02-20

**Authors:** Wenyan Sun, Bess Frost, Jiankang Liu

**Affiliations:** Center for Mitochondrial Biology and Medicine, The Key Laboratory of Biomedical Information Engineering of Ministry of Education, School of Life Science and Technology and Frontier Institute of Science and Technology, Xi'an Jiaotong University, Xi'an, China

**Keywords:** oleuropein, benefits

A “Mediterranean diet” rich in plant-based foods, fish, and olive oil, is associated with reduced risk of most aging-related diseases including metabolic syndrome and neurodegenerative disorders. Oleuropein, a polyphenolic compound enriched in olive oil and leaves of the olive tree, has attracted scientific attention in recent years because of a variety of reported health benefits. While the mechanisms by which oleuropein functions *in vivo* and *in vitro* have been investigated [1], more studies are needed to better understand oleuropein's protective mechanism of action and to develop oleuropein as a therapeutic. Oleuropein and its metabolite, hydroxytyrosol, have powerful antioxidant activity, which might be responsible for some of olive oil's antioxidant, anti-inflammatory, and disease-fighting activities.

Oleuropein is best known for its blood pressure-lowering effect. When administered via intraperitoneal or intravenous injections, oleuropein significantly reduces systolic and diastolic blood pressure in animal models. The ability of oleuropein to lower blood pressure may justify the traditional use of olive leaf in the treatment of mild to moderate hypertension. Our recent study provides significant insight into the mechanism whereby oleuropein reduces blood pressure. We find that oleuropein protects the hypothalamus from oxidative stress by improving mitochondrial function through activation of the Nrf2-mediated signaling pathway [2]. These effects are evident when supplementation occurs either before or after the onset of hypertension, suggesting that oleuropein is a promising strategy to both prevent and treat high blood pressure.

Beyond hypertension, oleuropein has been shown to have cardioprotective, anti-inflammatory, antioxidant, anti-cancer, anti-angiogenic and neuroprotective functions, and thus may be of therapeutic potential for a variety of human disorders. Oleuropein reduces oxidative damage in the substantia nigra of aged rats, a region of the brain that is most affected by neurodegeneration in Parkinson's disease [3]. Oleuropein prevents the toxic aggregation of both amyloid beta and tau, proteins that are involved in Alzheimer's disease [4, 5]. Of relevance to cancer, oleuropein is a potent inhibitor of human epidermal growth factor receptor 2, a protein that is frequently overexpressed in breast cancer cells [6], and exerts a chemopreventative effect on colitis-associated colorectal cancer in mice [7]. Mechanistic studies implicate autophagy and inhibition of the mammalian target of rapamycin (mTOR) in the protective effects of oleuropein [8]. Oxidative stress and deregulation of the mTOR pathway is a common theme among neurodegeneration, cancer, diabetes, and physiological aging, suggesting that the protective effects of oleuropein in various disorders may occur through a shared molecular mechanism.

Despite the many benefits of oleuropein, considerations about detrimental effects in certain individuals should not be neglected. Oleuropein may exacerbate low blood pressure in individuals who already have low blood pressure. Oleuropein could also interact with other pharmaceutical drugs that are designed to lower blood pressure or regulate diabetes. Patients should consult their physician before use and keep their physician informed of each medication being used in order to avoid additive or antagonistic drug interactions and side effects.

The unexpected benefits and unique properties of oleuropein provide ample rationale for continued use and study of this key component of the Mediterranean diet to promote human health. Future studies are needed for a more comprehensive understanding of the cellular networks involved in the diverse protective effects of oleuropein.

## References

[1] Mar SH (2010). Sci Pharm.

[2] Sun W (2017). Neuropharmacology.

[3] Sarbishegi M (2014). Iran. Biomed. J.

[4] Luccarini I (2014). Neurosci Lett.

[5] Daccache A (2011). Neurochem. Int.

[6] Fayyaz S (2016). Curr Top Med Chem.

[7] Giner E (2016). Mol Nutr Food Res.

[8] Rigacci S (2015). Oncotarget.

